# Locking system strengthened by biomimetic mineralized collagen putty for the treatment of osteoporotic proximal humeral fractures

**DOI:** 10.1093/rb/rbx016

**Published:** 2017-07-21

**Authors:** Cheng Peng, Hai-Peng Wang, Jia-Hua Yan, Tian-Xi Song

**Affiliations:** 1 Department of Orthopaedics, Jing’an District Centre Hospital, Jing’an Branch, Huashan Hospital Affiliated to Fudan University, Shanghai 200040, China; 2 Beijing Allgens Medical Science and Technology Co., Ltd., Beijing 100176, China

**Keywords:** mineralized collagen putty, osteoporosis, proximal humeral fracture, locking system

## Abstract

The current study is to observe the effect of the locking system strengthened by biomimetic mineralized collagen putty for the treatment of senile proximal humeral osteoporotic fractures. From January 2012 to December 2015, 80 cases of senile patients with osteoporotic proximal humeral fractures were randomly divided into an observation group and a control group, each group with a total of 40 cases. The control group was simply treated with locking plate. The observation group was treated with locking plate in combination with biomimetic mineralized collagen putty. The therapeutic effect thereby was observed. The excellent and satisfactory rate was 90% in observation group and was 72.5% in control group. The difference between the two groups was statistically significant (χ^2^ = 5.3312, *P *< 0.05). The fracture healing time was 11.82 ± 3.62 weeks in observation group and 19.78 ± 5.46 weeks in control group. The shoulder joint function score was 89.63 ± 8.12 in observation group and 76.92 ± 8.18 in control group. There was significant difference between the two groups (*t *= 7.1272; 12.7834, *P *< 0.05). The complication rate was 10% in the observation group and 32.5% in the control group (χ^2^ = 7.3786, *P *< 0.05). Locking system strengthened by biomimetic mineralized collagen putty has advantages such as accelerating healing of senile proximal humeral fracture, improving the therapeutic effect, reducing the complications. As one of the optimal internal fixation method, it provides a new option for better treatment of senile osteoporotic fracture.

## Introduction

Proximal humerus is one of the most common fracture sites in the elderly, incidence of which is related to violence and osteoporosis [1]. Osteoporotic fracture seriously threatens the physical and mental health of the elderly. With aged tendency of population, the incidence rate currently increases [2, 3]. Lack of effective treatment, many people resort to attempt like ‘repairing bone’, ‘fabricating bone’, ‘reinforcing bone’ and enhanced internal fixation, to pursue the so-called ‘biological osteosynthesis’. Presently there is of a wide variety of choices to treat this kind of fracture, but the effective and the ineffective are intermingled [4, 5].

The autologous bone granted the best curative effect, hence regarded as the golden standard of bony filling materials. However, it is likely to cause severe complications in the donor site, let alone its limited availability [6, 7]. Allogeneic bone grafting is an excellent succedaneum of autograft. But allograft is accompanied with risk of immune rejection, infection and transmitted diseases [8]. The bioceramic made of hydroxyapatite has modest capability of inducing osteogenesis, but is somewhat unsuitable for being injected into irregular defects [9, 10]. Calcium phosphate is a slightly soluble substance which is degraded by dissolution of the interstitial fluid. Degradation of calcium phosphate is usually too long for clinical application [11]. On the other hand, calcium sulfate is a slightly soluble substance which is degraded too quickly to maintain bone structure [12]. Both of the calcium phosphate and calcium sulfate has limited ability to guide osteogenesis.

Mineralized collagen putty is a novel form of artificial bone graft possessing biomimetic composition and microstructure similar to the natural bone tissues. It is prepared in a process called *in vitro* biomimetic mineralization technology with U.S.A. patent (No. 6887488). The injectable bone cement was designed on base of nano- hydroxyapatite/type I collagen fibril composites [13]. It has been approved by the Chinese Food and Drug Administration (CFDA Certification No: 20143462075) and US governments (FDA Certification No: K141725). They are biodegradable with excellent biological histocompatibility and osteointegration. The mineralized collagen putty is provided as porous blocks, and is able to become paste form after being immersed in saline or blood and pinch for a about 2 minutes. Such paste is injectable and moldable, thus suitable for many orthopaedic applications, such as filling irregular bone defects, carrying drugs, gluing crushed bones for bone fractures, and so on [14].

From January 2012 to December 2015, the research group selected appropriate patients with proximal humeral fractures for the treatment, applying anatomic locking plates with (40 cases as observation) or without (40 cases as control) biomimetic mineralized collagen putty. Curative effect and complication rate between the two groups were comparatively analyzed. The result showed that biomimetic mineralized collagen putty can strengthen the curative effect of locking plate in treating senile proximal fracture. Besides, this novel compound therapeutics could accelerate fracture heal, minimizing complication and improve the therapeutic effect. The report is presented as follows.

## Materials and methods

### Patient data

(1) Inclusion criteria for case selection were as the followings: Osteoporosis standard set by World Health Organization (WHO) [15] and *Diagnostic criteria of osteoporosis: year 2000 revision* [16] which was adopted by Committee of Osteoporosis of the Gerontology Society of China was referred to formulate the diagnostic criteria.

(2) Criteria for case exclusion were as the followings: 1, Case cannot meet the diagnostic criteria and inclusion criteria; 2, Patients have been undergoing long-term use of other relative drugs without possibility to stop the medication immediately; 3, Patients are under the influence of hyperparathyroidism, osteomalacia, chronic rheumatoid arthritis, multiple myeloma, secondary osteoporosis; 4, Patients are under the age of 40 or over the age of 90, pregnant or lactating women, person with allergies, mental illness, deformity in late stage, disability, or incapacitated; 5, Patients are complicated with serious primary diseases of heart, brain, liver, kidney, lung and hematopoietic system; 6, Patients participate in other clinical trials.

(3) The cases in the following situations would be rejected from experiment even after inclusion: 1, Patients didn’t comply with provisions of the follow-up, or couldn’t provide complete information on the efficacy or side effect, or couldn’t judge the potency or efficacy of the therapy; 2, Patients suspended clinical trial because of other diseases; 3, Patients lost their case report.

(4) Grouping method: Using random sampling number table, patients were randomly divided into observation group and treatment group with 40 cases in each group. There was no significant difference in age, gender and state of fracture between the two groups (*P* > 0.05) [17].

A total of 80 cases were selected, including 32 males and 48 females, from 60 to 88 years of age (with an average age of 74.3 years), 32 cases on the left and 48 cases on the right. According to the Neer classification, 2-part fractures in 15 cases, 3-part fractures in 36 cases, and 4-part fractures in 29 cases. Nine of the cases were complicated with shoulder joint dislocation, 28 cases with hypertension, and 31 cases with diabetes.

### Operation methods

**Figure 1.**
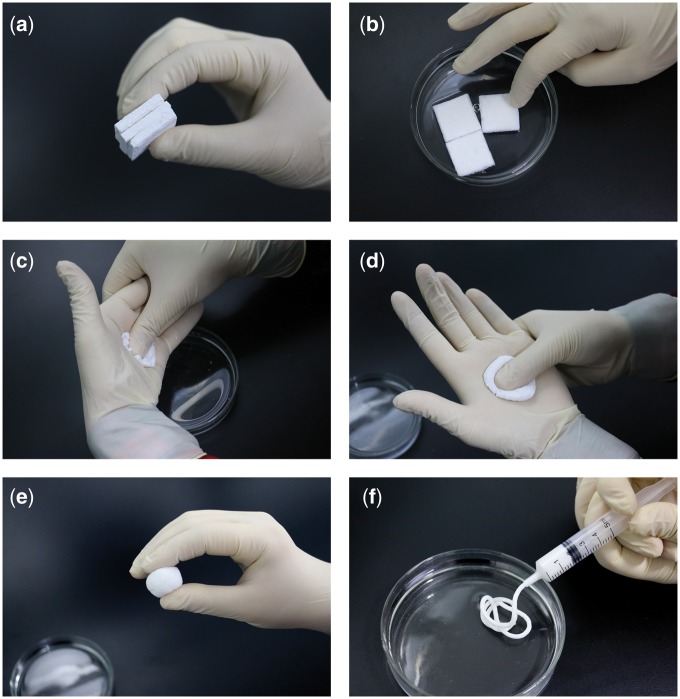
Mineralized collagen putty was intraoperatively prepared. (**a**) Mineralized collagen putty blocks were taken from the package. (**b**) They were immersed into normal saline for about 30 seconds. (**c**) Liquid was extrude from the putty. (**d**) The putty was malaxated and kneaded by a thumb in the other palm. (**e**) Malaxation and knead were continued for about 2 minutes to form a paste. (**f**) The mineralized collagen putty paste could be squeezed through an injector

**Figure 2.**
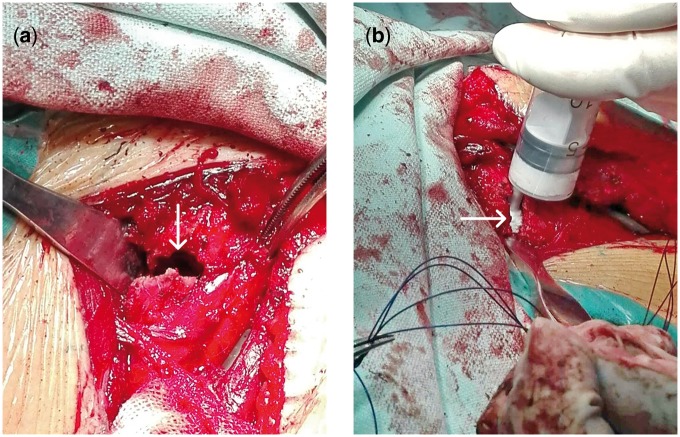
In the observation group, segmental bone defects of humeral head were filled with mineralized collagen putty. (**a**) The fractured humeral segments were distracted to show the defect (noted by arrow). (**b**) The mineralized collagen putty was injected into the defect (noted by arrow)

**Figure 3.**
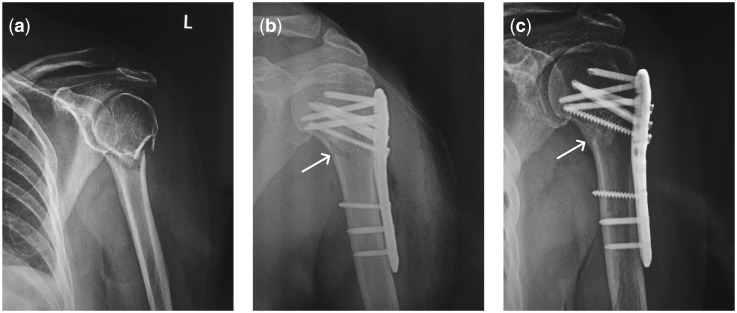
A case of 76 years old female patient diagnosed as comminuted fractures of the left proximal humerus. (**a**) An X-ray film taken before surgery, in which the comminuted fracture of the surgical neck of humerus could be seen. (**b**) Radiograph was taken one day after operation for postoperative reexamination. All fractures showed good contraposition and alignment with satisfactory internal fixation. (**c**) Radiograph was taken three months after operation. Fracture appeared healed with good union. There was neither osteonecrosis nor fracture of humeral head. The internal fixation nails were neither pulled out nor loosened

**Figure 4.**
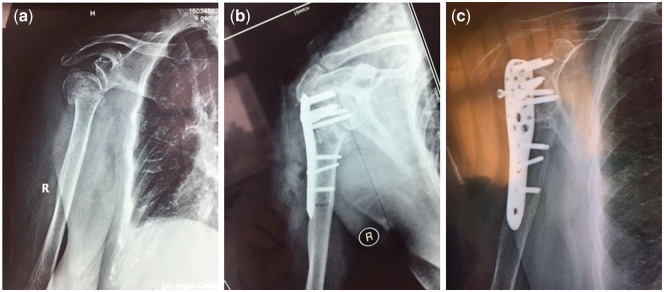
A case of 85 years old female patient diagnosed as comminuted fractures of the right proximal humerus. (**a**) An X-ray film taken before surgery, in which the comminuted fracture of the surgical neck of humerus could be seen. (**b**) Radiograph was taken one day after operation for postoperative reexamination. All fractures showed good contraposition and alignment with satisfactory internal fixation. (**c**) Radiograph was taken three months after operation. The screw loosened and pierced out of the locking plate fixation. Humeral head underwent varus deformity and absorption

Mineralized collagen putty (produced by Beijing Allgens Medical Science and Technology Co., Ltd.) was intraoperatively prepared as follow steps: 1, take the mineralized collagen putty blocks from the package (Fig. 1a); 2, immerse them into saline for about 30 seconds (Fig. 1b); 3, extrude water from the putty (Fig. 1c); 4, pinch the putty by a thumb in the other palm (Fig. 1d); 5, pinch for about 2 minutes to form a paste (Fig. 1e); 6, the mineralized collagen putty paste could be implanted by using an injector (Fig. 1f).

Surgeries were operated by the same surgeon. After anesthesia, the patient lay in the supine position with shoulder pad. With anterior arc incision, the surgical operation was approached through the gap between deltoid and pectoralis major muscle. Cervical blood vessels were protected during operation. After removing the embedded soft tissue from broken ends, surgeon inserted a 3 mm Kirschner wire into the proximal end of fracture [18]. The surgeon percutaneously reduced the displaced proximal humeral head, to make the humeral head, especially the greater and lesser tuberosity humerus and humeral metaphysis, to achieve the best possible reduction. Kirschner wire and PDS thick line were temporarily fixed. To confirm correct reduction of the fracture, foci were examined by C-arm X-ray machine fluoroscopy in the anteroposterior and reverse lateral transthoracic perspectives. The anatomical locking plate in the proximal humerus was put at the front of the insertion of deltoid, the proximal of which was placed about 5 mm behind the intertubercular sulcus and 5 mm below the proximal of greater tuberosity humerus. The first screw was placed into the sliding hole in humeral shaft, while plate height was adjusted under X-ray guidance. In the observation group, segmental bone defects of humeral head were filled with biomimetic mineralized collagen putty (Fig. 2). The control group was not treated with bone grafting. To increase the stability, as many as possible locking screws were driven into the proximal end of fracture and 3 ∼ 4 locking screws were driven into the distal end. C-arm X-ray machine fluoroscopy was used again to ensure fracture reduction, as well as position and length of the screw. Make sure there was no obstacle in shoulder joint when it was exposed to passive motion. Make sure there was no loosening of internal fixation. Injuries to rotator cuff and joint capsule were repaired by thick absorbable suture. The crushed bone blocks which still connected to soft tissue were fixed to the suture hole at the proximal end of the plate. After the wound was debrided, the incision was closed by layer-by-layer suture.

### Postoperative treatment

Required by the National Health and Family Planning Commission of the PR China, type I incision (using implants) should routinely use prophylactic antibiotic for 24 hours. Given postoperative analgesia under the guidance, patients can perform active and passive functional exercise of shoulder joints in painless condition. Common diseases of internal medicine were conventionally diagnosed and treated. All these pains were taken to help the patients safely get through perioperative period. The patients were treated with conventional anti-osteoporosis measures. Radiographs were taken every 4 weeks to understand the position of screw and state of fracture healing, so as to guide functional exercise of shoulder joint.

### Statistical methods

All the data were analyzed using statistical software SPSS13.0. The enumeration data were shown as rate (%), while measurement data were shown as “$\bar{x}$ ± s”. *P* < 0.05 means significant difference.

## Results

All the 80 cases were followed up for 14–24 months, with an average of 15.6 months. Most of the fracture healed 3–7 months after surgical operation. Function scores in the last follow-up after fracture healing were analyzed using the American Shoulder and Elbow Surgeons (ASES) score evaluation system [19]. Among the full score of 100 points, both pain and daily life ability accounted for 50 points. The score as high as 90 ∼ 100 points was deemed to be excellent, 80 ∼ 89 points to be good, 7 0 ∼79 points to be tolerable, less than 70 points to be poor. The excellent/good rate in the observation group was 90%, contrasted with 72.5% in the control group with significant difference (*P *<* *0.05). Comparison of the therapeutic effects between the two groups was presented in Table 1.

**Table 1.** Comparison of surgical treatment effect between the two groups

Group	No. of cases	Excellent [rate (%)]	Good [rate (%)]	Tolerable [rate (%)]	Poor [rate (%)]	Excellent/good [rate (%)]
Observation group	40	26 [65.0%]	10 [25.0%]	3 [7.5%]	1 [2.5%]	36 [90.0%]
Control group	40	21 [52.5%]	8 [20.0%]	9 [22.5%]	2 [5.0%]	29 [72.5%]^a^

Note: Compared with the control group ^a^, χ^2^ = 5.3312, *P* < 0.05.

The complication incidence was evaluated between the two groups. In the observation group, there were one case of osteonecrosis of humeral head, two cases of loosening of the internal fixation and one case of shoulder inversion observed after surgical operation. In the control group, there were three cases of osteonecrosis of humeral head, five cases of loosening of the internal fixation and three cases of shoulder inversion observed after surgical operation. The complication incidence was 10.0% (4 out of 40) in the observation group and 27.5% (11 out of 40) in the control group. The complication incidence of the observation group is significantly higher than that of the control group (*P *<* *0.05). The detailed comparison of complication incidence was summarized in Table 2.

**Table 2.** Comparison of operative complications between the two groups

Group	No. of cases	Osteonecrosis of humeral head [cases (%)]	Internal fixation loosening [cases (%)]	Shoulder inversion [cases (%)]	Total incidence [rate (%)]
Observation group	40	1 [2.5%]	2 [5.0%]	1 [2.5%]	4 [10.0%]
Control group	40	3 [7.5%]	5 [12.5%]	3 [7.5%]	11 [27.5%]

Note: Compared with the control group ^a^, χ^2^ = 7.3786, *P* < 0.05.

In the observation group, fracture healing time was 11.82 ± 3.62 weeks and shoulder joint function score was 89.63 ± 8.12 points, contrasted with 19.78 ± 5.46 weeks and 76.92 ± 8.18 points in control group. Although observation group performed better than control group, there is no statistical significance between them. The detailed comparison of these two indices was summarized in Table 3.

**Table 3.** Comparison of fracture healing time and shoulder joint function score between the two groups

Indices/Group	Fracture healing time (weeks)	Shoulder joint function score (points)
Observation group	11.82 ± 3.62	89.63 ± 8.12
Control group	19.78 ± 5.46	76.92 ± 8.18

There were typical cases in both groups which presented intuitive comparison. For example, 76 years old female patient diagnosed as comminuted fractures of the proximal left humerus was treated in the observation group. The authors evaluated X-ray observation of patients before and after treatment with internal fixation and biomimetic mineralized collagen putty. X-ray film taken before surgery showed comminuted fracture of the surgical neck of humerus (Fig. 3a). One day after operation, all fractures showed good contraposition and alignment on the X-ray film (Fig. 3b). Bone defect was implanted with biomimetic mineralized collagen putty, along with satisfactory internal fixation. Three months after operation, the fracture appeared healed with good fixation and union. There was neither osteonecrosis of humeral head nor fracture, proving excellent osteogenesis. The patient was free of pull out or loosening of internal fixation because of increased bone mass and rapid healing of the fracture. On the other hand, a case of 85 years old female patient diagnosed as comminuted fractures of the right proximal humerus showed different prognosis in the control group. An X-ray film taken before surgery showed comminuted fracture of the surgical neck of humerus (Fig. 4a). Radiograph was taken one day after operation for postoperative reexamination. All fractures showed good contraposition and alignment with satisfactory internal fixation (Fig. 4b). Radiograph was taken three months after operation. The screw found to be loosened and pierced out of the locking plate fixation. Humeral head poorly healed and underwent varus deformity and absorption (Fig. 4c).

## Discussion

There have been difficulties in clinical treatment of senile osteoporotic fracture, which is frequently accompanied by comminuted fracture, easily leading to bone defect. In this case, fracture reduction and fixation is very difficult, along with poor rigidity and difficulties in implant or internal fixation. Fracture healing and callus maturation are delayed, accompanied with impairment of bone healing quality, mechanical strength, early full weight-bearing and rehabilitation of limb function [20–22]. Mass and quality of bone can hardly improve in the short term, with significantly increased risk of nonunion and refractures. Despite the continual evolution of locking plate techniques and designs to increase the stability of osteoporotic fractures fixation, so far there are still conditions such as loosening of lock pin, excision of locking plate, fracture of internal fixation and osteonecrosis of humeral head [23, 24]. Locking plate cannot be just installed and left alone. Some doctors administered bone cement to optimize purchase of screw. Although its short-term effect is satisfactory, bone cement is disincentive to bone healing. Therefore, bone formation is somewhat difficult on account of the cauterizing effect of bone cement on the surrounding tissues [25]. Although self-ilium graft conduces to good prognosis, it is limited by available bone mass. Complications caused by wound of increased donor sites must be considered in the clinical practice [26]. Osteoporotic fracture is still a difficult point in clinical practice, which has aroused widespread interest from orthopedic circle and presents the future directions and problems worthy of studying for orthopedists. Therefore, finding an effective measure to treat osteoporotic fracture is the problem that encounters orthopedists. The development of nanotechnology clears a path to solve the problem.

Biomimetic artificial mineralized collagen is a kind of artificial bone graft which mimics composition and microstructure of human natural bone [27–29], and has been widely applied in clinical area of orthopedics, stomatology, neurosurgery and so on [30–33]. Its excellent curative effect in bone defect reparation has been proven in more than 200 000 cases of clinically application. However, similar to traditional allograft and artificial bone repair materials, the mineralized collagen bone grafts were clinically supplied with fixed shape and size, such as particle, block and strip. Such shapes of the bone graft materials limited their application in filling and repairing for irregular bone defect. Artificial mineralized collagen putty is a new formulation of biomimetic mineralized collagen bone repair material. The material is provided in the form of porous blocks. After being mixed with saline or blood and pinched for about 2 minutes during the surgery, it will form a paste which can be voluntarily shaped and used by injection. It not only meets the clinical needs of filling and repairing irregular bone defect in clinical practice of orthopedics, but could also be used for bonding bone fragments. It makes full use of autologous bone in fracture site, expediting fracture healing and new bone regeneration in bone defect. It is proven by animal experiments that the artificial mineralized collagen degrades in step with new bone regeneration [20a]. This quality of matching and synchronization endow it with excellent osteogenetic activity.

This group study, we used biomimetic mineralized collagen putty as bone grafting material, combined with the locking plate in the treatment of senile osteoporotic proximal humeral fracture. Bone defect in 40 cases of senile osteoporotic fracture was packed with biomimetic mineralized collagen putty. Observed by X-ray and clinical manifestations, fracture healing of the 40 cases of senile osteoporotic fracture was shown to be expedited, with fracture healed and bone graft material absorbed within 12 weeks. Shoulder joint showed better function than the control group while the complication rate is lower than the control group.

## References

[1] Maier D, Jager M, Strohm PC Treatment of proximal humeral fractures–a review of current concepts enlightened by basic principles. Acta Chir Orthop Traumatol Cech 2012;79:307–16. 22980928

[2] aJakob F, Seefried L, Schwab M. Age and osteoporosis. Effects of aging on osteoporosis, the diagnostics and therapy. Internist (Berl) 2014;55:755–61. 24903137 10.1007/s00108-014-3468-z

[3] bLiu LK, Lee WJ, Chen LY, Association between frailty, osteoporosis, falls and hip fractures among community-dwelling people aged 50 years and older in Taiwan: results from I-lan longitudinal aging study. PLoS One 2015;10:e0136968. 26348034 10.1371/journal.pone.0136968 PMC4562637

[4] aZhan BL, Ye Z. Hook-screw combination to treat for the unstable Hangman's fracture. Zhongguo Gu Shang 2009;22:830–1. 20084939

[5] bHuang ZJ, Guan JZ, Xu YJ, Clinical research on zishengukang pill (see text) used to treat delayed union of fracture. J Tradit Chin Med 2011;31:189–91. 21977860 10.1016/s0254-6272(11)60039-3

[6] aChou LB, Mann RA, Coughlin MJ, Stress fracture as a complication of autogenous bone graft harvest from the distal tibia. Foot Ankle Int 2007;28:199–201. 17296139 10.3113/FAI.2007.0199

[7] bDe Riu G, Meloni SM, Raho MT, Delayed iliac abscess as an unusual complication of an iliac bone graft in an orthognathic case. Int J Oral Maxillofac Surg 2008;37:1156–8. 18775644 10.1016/j.ijom.2008.07.018

[8] Regel G, Sudkamp NP, Illgner A 15 years allogeneic bone transplantation. Indications, treatment and results. Unfallchirurg 1992;95:1–8. 1566088

[9] aLee HR, Kim HJ, Ko JS, Comparative characteristics of porous bioceramics for an osteogenic response in vitro and in vivo. PLoS One 2013;8:e84272. 24391927 10.1371/journal.pone.0084272 PMC3877265

[10] bIzci Y, Secer HI, Ilica AT, *.* The efficacy of bioceramics for the closure of burr-holes in craniotomy: case studies on 14 patients. J Appl Biomater Funct Mater 2013;11:e187–96. 22798240 10.5301/JABFM.2012.9252

[11] Cai S, Zhai Y, Xu G Preparation and properties of calcium phosphate cements incorporated gelatin microspheres and calcium sulfate dihydrate as controlled local drug delivery system. J Mater Sci Mater Med 2011;22:2487–96. 21894539 10.1007/s10856-011-4432-2

[12] Nilsson M, Zheng MH, Tagil M. The composite of hydroxyapatite and calcium sulphate: a review of preclinical evaluation and clinical applications. Expert Rev Med Devices 2013;10:675–84. 24053255 10.1586/17434440.2013.827529

[13] Liu X, Wang XM, Chen Z Injectable bone cement based on mineralized collagen. J Biomed Mater Res B Appl Biomater 2010;94:72–9. 20336741 10.1002/jbm.b.31625

[14] Xie BG, Wang XD, Wang H Releasing profile of antibiotics from a novel mineralized collagen putty with quantifiable drug loading. Mater Technol 2015;30:B216–B22.

[15] Kanis JA, Melton LJ III, Christiansen C The diagnosis of osteoporosis. J Bone Miner Res 1994;9:1137–41. 7976495 10.1002/jbmr.5650090802

[16] Orimo H, Hayashi Y, Fukunaga M Diagnostic criteria for primary osteoporosis: year 2000 revision. J Bone Miner Metab 2001;19:331–7. 11685647 10.1007/s007740170001

[17] Eghbali-Babadi M, Ghadiriyan R, Hosseini SM. The effect of saline lock on phlebitis rates of patients in cardiac care units. Iran J Nurs Midwifery Res 2015;20:496–501. 26257807 10.4103/1735-9066.161006 PMC4525350

[18] Roberts VI, Komarasamy B, Pandey R. Modification of the Resch procedure: a new technique and its results in managing three- and four-part proximal humeral fractures. J Bone Joint Surg Br 2012;94:1409–13. 23015570 10.1302/0301-620X.94B10.28692

[19] King GJ, Richards RR, Zuckerman JD A standardized method for assessment of elbow function. Research Committee, American Shoulder and Elbow Surgeons. J Shoulder Elbow Surg 1999;8:351–4. 10472009 10.1016/s1058-2746(99)90159-3

[20] aNewman JM, Kahn M, Gruson KI. Reducing postoperative fracture displacement after locked plating of proximal humerus fractures: current concepts. Am J Orthop (Belle Mead NJ) 2015;44:312–20. 26161759

[21] bVijayvargiya M, Pathak A, Gaur S. Outcome analysis of locking plate fixation in proximal humerus fracture. J Clin Diagn Res 2016;10:RC01–5. 10.7860/JCDR/2016/18122.8281 PMC5028477 27656515

[22] cHsiao CK, Tsai YJ, Yen CY, Intramedullary cortical bone strut improves the cyclic stability of osteoporotic proximal humeral fractures. BMC Musculoskelet Disord 2017;18:64. 28153021 10.1186/s12891-017-1421-8 PMC5290624

[23] aHettrich CM, Neviaser A, Beamer BS, Locked plating of the proximal humerus using an endosteal implant. J Orthop Trauma 2012;26:212–5. 22337487 10.1097/BOT.0b013e318243909c

[24] bIacobellis C, Berizzi A, Biz C, Treatment of proximal humeral fractures with reverse shoulder arthroplasty in elderly patients. Musculoskelet Surg 2015;99:39–44. 24917462 10.1007/s12306-014-0331-2

[25] Mazzantini M, Carpeggiani P, d'Ascanio A Long-term prospective study of osteoporotic patients treated with percutaneous vertebroplasty after fragility fractures. Osteoporos Int 2011;22:1599–607. 20661546 10.1007/s00198-010-1341-z

[26] Zhu L, Liu Y, Yang Z Locking plate fixation combined with iliac crest bone autologous graft for proximal humerus comminuted fracture. Chin Med J (Engl) 2014;127:1672–6. 24791873

[27] aLiao SS, Cui FZ, Zhang W, Hierarchically biomimetic bone scaffold materials: nano-HA/collagen/PLA composite. J Biomed Mater Res B Appl Biomater 2004;69:158–65. 15116405 10.1002/jbm.b.20035

[28] bCui F-Z, Li Y, Ge J. Self-assembly of mineralized collagen composites. Mater Sci Eng: R: Rep 2007;57:1–27.

[29] cQiu Z-Y, Cui Y, Tao C-S, Mineralized collagen: rationale, current status, and clinical applications. Materials 2015;8:4733–50. 28793468 10.3390/ma8084733 PMC5455477

[30] aYu X, Xu L, Cui FZ, Clinical evaluation of mineralized collagen as a bone graft substitute for anterior cervical intersomatic fusion. J Biomater Tissue Eng 2012;2:170–6.

[31] bKou J-M, Fu T-Y, Jia X-J, Clinical observations on repair of non-infected bone nonunion by using mineralized collagen graft. J Biomater Tissue Eng 2014;4:1107–12.

[32] cQiu Z, Zhang Y, Zhang Z, Biodegradable mineralized collagen plug for the reconstruction of craniotomy burr-holes: a report of three cases. Trans Neurosci Clin 2015;1:3–9.

[33] dFeng L, Zhang L, Cui Y, Clinical evaluations of mineralized collagen in the extraction sites preservation. Regener Biomater 2016;3:41–8. 10.1093/rb/rbv027 PMC4723274 26815224

